# Diagnostics for COVID-19: A case for field-deployable, rapid molecular tests for community surveillance

**DOI:** 10.4314/gmj.v54i4s.11

**Published:** 2020-12

**Authors:** Michael Frimpong, Yaw A Amoako, Kwadwo B Anim, Hubert S Ahor, Richmond Yeboah, Joshua Arthur, Justin S Dakorah, Delphine Gborgblovor, Samuel Akrofi, Phyllis Sekyi-Djan, Michael Owusu, Augustina A Sylverken, Tabea Binger, Richard O Phillips

**Affiliations:** 1 Kumasi Centre for Collaborative Research, Kwame Nkrumah University of Science and Technology, Kumasi, Ghana; 2 Department of Molecular Medicine, KNUST School of Medicine and Dentistry, Kwame Nkrumah University of Science and Technology, Kumasi, Ghana; 3 Department of Medicine, Komfo Anokye Teaching Hospital, Kumasi, Ghana; 4 AngloGold Ashanti Health Foundation, AngloGold Ashanti Obuasi Mine, Obuasi, Ghana; 5 Obuasi Health Directorates, Ghana Health Service, Obuasi, Ghana; 6 Public Health, Komfo Anokye Teaching Hospital, Kumasi, Ghana; 7 Customs Laboratory, Ghana Revenue Authority, Accra, Ghana; 8 Department of Theoretical and Applied Biology, Kwame Nkrumah University of Science and Technology, Kumasi, Ghana

**Keywords:** COVID-19, Polymerase Chain Reaction, Point-of-care test, SARS-CoV-2, Mobile Laboratory

## Abstract

Across the globe, the outbreak of the COVID-19 pandemic is causing distress with governments doing everything in their power to contain the spread of the novel coronavirus (SARS-CoV-2) to prevent morbidity and mortality. Actions are being implemented to keep health care systems from being overstretched and to curb the outbreak. Any policy responses aimed at slowing down the spread of the virus and mitigating its immediate effects on health care systems require a firm basis of information about the absolute number of currently infected people, growth rates, and locations/hotspots of infections. The only way to obtain this base of information is by conducting numerous tests in a targeted way. Currently, in Ghana, there is a centralized testing approach, that takes 4–5 days for samples to be shipped and tested at central reference laboratories with results communicated to the district, regional and national stakeholders. This delay in diagnosis increases the risk of ongoing transmission in communities and vulnerable institutions. We have validated, evaluated and deployed an innovative diagnostic tool on a mobile laboratory platform to accelerate the COVID-19 testing. A preliminary result of 74 samples from COVID-19 suspected cases has a positivity rate of 12% with a turn-around time of fewer than 3 hours from sample taking to reporting of results, significantly reducing the waiting time from days to hours, enabling expedient response by the health system for contact tracing to reduce transmission and additionally improving case management.

**Funding:**

Test kits were provided by AngloGold Ashanti Obuasi Mine (AngloGold Ashanti Health Foundation). The American Leprosy Mission donated the PCR machine, and the mobile laboratory van was funded by the Embassy of the Kingdom of the Netherlands (EKN). AAS, YAA was supported by (PANDORA-ID-NET RIA2016E-1609) and ROP supported by EDCTP Senior Fellowship (TMA2016SF), both funded by the European and Developing Countries Clinical Trials Partnership (EDCTP2) programme which is supported under Horizon 2020, the European Union.

## Introduction

The COVID-19 pandemic continues to ravage the populations of many countries around the world. This coronavirus infection first reported in Wuhan, China in December 2019 has now been designated as SARS-CoV-2.^1^ The disease has spread to many countries around the world with Ghana reporting its first laboratory confirmed case of COVID-19 on 12 March 2020. Ghana has recorded 28,989 cases with 153 deaths^2^ and the situation is rapidly evolving. The scale of the problem has necessitated calls for increased action by the government of Ghana.

The country's strategy for fighting the scourge of the disease has been focused on the three pillars of early identification and testing of cases, treatment of cases and enhanced contact tracing.^3^ This strategy requires a readily available, fast and accurate diagnostic testing method.

In Ghana, the Noguchi Memorial Institute for Medical Research (NMIMR) and the Kumasi Centre for Collaborative Research in Tropical Medicine (KCCR) have been the two main laboratories at the forefront of laboratory testing. When the country began seeing imported cases, the virology laboratories at NMIMR and the KCCR received and processed samples from the southern and northern zones of the country respectively.^4^ More recently, the Public Health Reference Laboratory (PHRL) at the Korle-Bu and some regional and institutional laboratories have been equipped to aid the testing efforts to improve the turnaround time for testing.^3^ Despite the relatively increased number of testing sites, there are still challenges with the laboratory confirmation process with many reports of delays in the release of test results.^5^ These centralized laboratories receive and process samples from different parts of Ghana. Delays in the transportation of samples as well as the high number of samples received at the laboratories have the potential to hamper the early confirmation of cases of COVID-19 with a knock-on effect on the tracing and isolation of case contacts. We report on an innovative diagnostic tool on a mobile laboratory platform that has been deployed to accelerate the COVID-19 diagnostic process from days to hours, enabling expedient response by the health system for contact tracing to reduce transmission of the infection.

## Methods

### Set up of mobile laboratory

**Figure 1:**
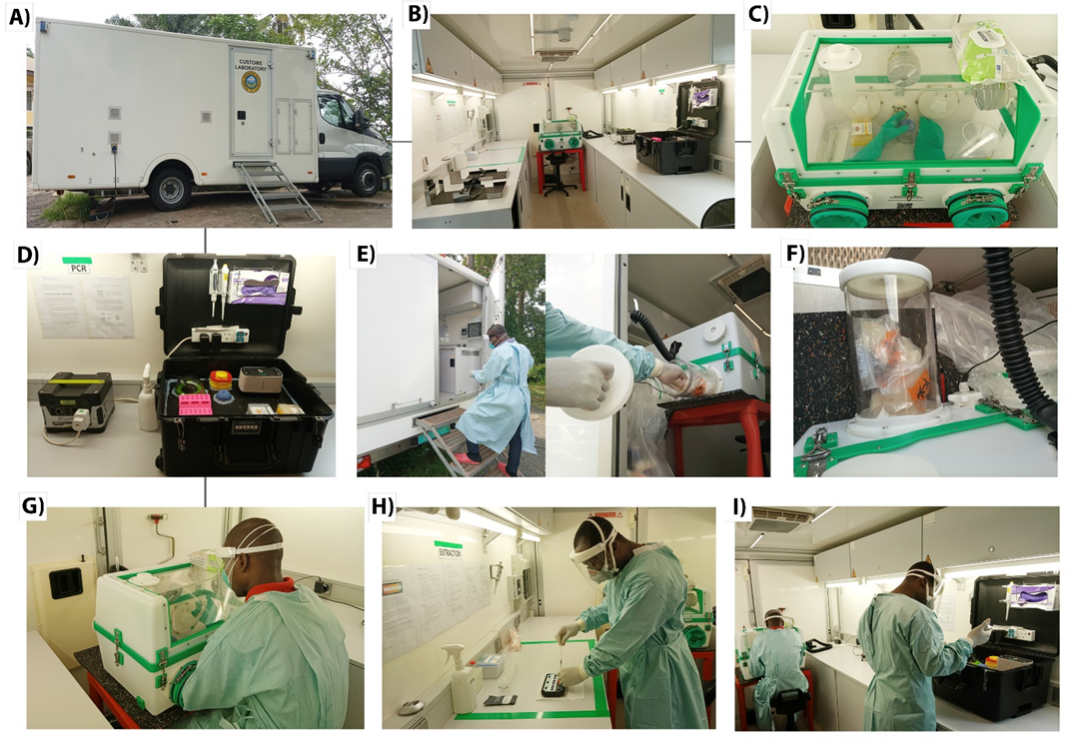
SARS CoV-2 Mobile laboratory. A) Mobile Van laboratory, B) Inside the van, a complete setup for COV1ID-19 testing C) Biosafety Level 3 (BSL3) Glove-box, the workspace inside the glovebox contained a vortex mixer (IKA lab dancer, Germany), a box of 100–200 µl sterile filter tips with an automatic micropipette, a rack of 2.0 ml cryovials and a marker pen. D) Mobile suitcase containing all the reagents and equipment (a mini-Centrifuge, a vortex mixer, 2 automatic micropipettes and Franklin's qPCR thermal Cycler. Via the back outlet directly into the glovebox. E) Samples delivery into van, F) samples awaiting processing, G) Sample processing and inactivation, H) Extraction of viral RNA using the Biomeme M1 sample prep cartridge RNA 2.0 kit, I) Amplification of viral RNA using the Biomeme SARS-CoV-2 Go-Strip kits on Franklin's qPCR thermocycler.

A mobile van laboratory belonging to the Ghana Customs Laboratory was repurposed for COVID-19 testing. The van contains a mini refrigerator/freezer compartment for sample storage, a system for producing deionized water, cabinets for storing consumables, and a handwashing system. It has an in-built generator in the absence of electricity and can also be plugged into the national grid where power is available (Figure 1A and 1B). In repurposing the laboratory, the refrigeration system was used for the storage of reagents, samples and RNA extracts. A biosafety level three (BSL3) glovebox (Ultra glovebox, Koennecke, Berlin, Germany) (Figure 1C) was also fitted into the laboratory to meet the requirement for SARS CoV-2 testing. The glovebox was specifically for sample preparation, inactivation and extraction. A mobile suitcase laboratory for amplification using the Biomeme FranklinTM Three-9 qPCR thermocycler was also installed in the laboratory.

The mobile suitcase laboratory was developed with a pelican case (Pelican 1607 Air) of dimension 56 cm × 45.5 cm × 26.5 cm 6,7 (Figure 1D). This case is stuffed with foams to act as a shock absorber during transportation or the operation of the equipment. A PVC carpet was attached to the foam, into which indents were cut and sealed to make the case watertight. These indents created housed a mini centrifuge and pulse vortex, pipette tips and a waste bin. The lid of the pelican case contains gloves, automatic micropipettes, marker and an electricity extension board. A power pack (Yeti 150 set, GOAL ZERO, South Bluffdale, UT, USA) was also provided as the main source of the energy for the suitcase laboratory.

### Validation of Biomeme SARS-CoV-2 assay

Before the deployment of the mobile laboratory using the Biomeme SARS-CoV-2 assay in the field, we validated the Biomeme SARS-CoV-2 Go Strip with three RNA extracts.

The M1 sample Prep Cartridge RNA 2.0 was also validated with ten samples. All RNA extracts and samples were processed with in-house qPCR assay for diagnosis of COVID-19.

### Sample collection and transportation

Between 7th July and 30th July 2020, samples were obtained from patients with suspected COVID-19 from the AngloGold Ashanti Health Foundation Hospital in Obuasi in the Ashanti Region of Ghana. Recruitment of cases at these facilities was done according to the Ghana National Surveillance Strategy protocol. Briefly suspected COVID-19 cases were defined as individuals presenting with fever (>38°C), or a history of fever and symptoms of respiratory tract illness such as cough, shortness of breath, or individuals in close contact with a person who is under investigation or confirmed for COVID-19. Nasopharyngeal and or oropharyngeal swabs were obtained with flocked swabs (Copan Group, Brescia, Italy) and kept in viral transport media in 1.5ml tubes (Eppendorf, Regensburg, Germany) and transported immediately for confirmation at Mobile van laboratory.

### Laboratory diagnosis of suspected Covid-19 patients in the field

Four biologists were trained in biosafety protocols for SARS-CoV-2 molecular diagnosis to man the laboratory. After a full day of training at KCCR, one of the leading COVID-19 testing laboratories in Ghana, the mobile laboratory was deployed to Obuasi to serve both the Municipality and Obuasi East District.

The mobile vehicle was stationed in AngloGold Ashanti Health Foundation, Obuasi Municipality, Ghana (Obuasi had been designated as a point of need/ epicentre during the outbreak by the Ghana Health Service), to minimise travel distance and the duration of sample arrival at the mobile laboratory. Sample containers on arrival at the laboratory were decontaminated with 0.1% chlorine solution, cleaned and samples placed in the Glovebox. In the Glovebox, samples were vortexed to mix and aliquoted into 2 mL cryovial. 200 µL of samples was added to Biomeme Lysis Buffer (BLB) in the M1 sample prep cartridge RNA 2.0 and incubated for 10 minutes to inactivate the samples in the glovebox. The initial phase of the extraction, (lysis and binding) was carried out in the Glovebox, by attaching a sample prep column into the red section of the cartridge and the BLB pulled all the way up the syringe and pumped back down again. This procedure of lysis and binding is repeated ten times after which the cartridge and the sample prep column are brought out of the Glovebox. Subsequent steps of viral RNA extraction (protein, salt, dry wash, drying and elution) were performed according to the manufacturer's procedure on the bench. Twenty microlitres of RNA extract was added to the Biomeme SARS-CoV-2 Go-Strips and incubated in the Franklin™ Real-Time PCR thermocycler. The operation of the machine and cycling conditions of the SARSCoV-2 assay was done according the manufacturer's protocol and data of complete run were obtained via the Biomeme Go App. To ensure that the quick testing procedure within the mobile laboratory did not affect its performance as a diagnostic test for COVID-19, 20% of samples were retested in the reference laboratory at KCCR using an established test procedure.

### Data Analysis

Microsoft Excel 2016 was used for data management. Descriptive statistics were used to obtain general descriptive information such as median and interquartile ranges from the data. Simple proportions were calculated to determine the positive rate in each subgroup.

### Ethical Statement

Ethical approval for this study was obtained from the Committee on Human Research, Publication and Ethics (CHRPE/AP/462/19) School of Medical Sciences, Kwame Nkrumah University of Science and Technology and the Institutional Review Board of the Ghana Health Service (GHS-ERC087/03/20). Permission was also sought from Obuasi Health Directorates to conduct the study.

## Results

Before deployment of the mobile van laboratory, the Biomeme SARS-CoV-2 assay which was to be used in the mobile laboratory was evaluated and validated with 13 archived COVID-19 suspected samples. The assay had 100% agreement with the in-house qPCR assay for diagnosis of Covid-19 as shown in Table 1.

**Table 1:** Results of assay validation

Sample ID	Type of sample	Central lab PCR result	Biomeme PCR result
**1**	RNA extract	-	-
**2**	RNA extract	+	+
**3**	RNA extract	+	+
**4**	Clinical sample	+	+
**5**	Clinical sample	-	-
**6**	Clinical sample	-	-
**7**	Clinical sample	-	-
**8**	Clinical sample	-	-
**9**	Clinical sample	+	+
**10**	Clinical sample	+	+
**11**	Clinical sample	+	+
**12**	Clinical sample	-	-
**13**	Clinical sample	-	-

The mobile laboratory was easy to transport to the point of need (in the field). The setup of the laboratory including the assembly of the glovebox, the suitcase laboratory together with donning of PPE took approximately 30 minutes and the team was ready to receive samples for processing. Once samples were received, the inactivation step including the lysis and extraction of viral nucleic acid to the column was performed in the glovebox for up to 7 samples in 20 minutes, while the RNA purification step including the various wash steps and elution on the bench (Figure 1H) needed about 10 minutes.

Since all the reagents needed for PCR has already been lyophilized in a dried-format, Ready-to-Go (SARS-CoV-2 Go-Strips), the amplification step including pipetting, mobile App connection and login, PCR protocol setup lasted about 65 minutes after which the results were ready to be analyzed and subsequently reported to the right authority for the necessary action.

To date, a total of 74 nasopharyngeal swab samples from suspected Covid-19 cases and their contacts have been tested. Of this number, 20 (33%) were symptomatic while 54 (67%) were asymptomatic. In all 9 (12%) were positive for COVID-19 (Table 2). Of these 9 positive cases, 4 were samples from asymptomatic patients and 5 were from persons with symptoms consistent with the case definition of suspected COVID-19.

**Table 2:** Demographics of suspected covid-19 cases and their contacts

Characteristics	Frequency N (%)	No. Positive (% positivity)
**Gender**		
**Male**	46 (62)	3 (6.5%)
**Female**	28 (38)	6 (21%)
**Age in years Median** **(IQR)**	34 (29–43)	
**Age Category (year)**		
**≤ 15**	3(3.3)	0
**16–30**	19 (30.0)	5 (26%)
**31–45**	35 (41.7)	3 (9%)
**46–60**	12 (18.3)	0
**≥ 60**	5 (5.0)	1 (20%)
**Cases**		
**Symptomatic**	20 (33.3)	4 (20%)
**Asymptomatic**	54 (66.7)	5 (9%)
**Total tested**	74	9 (12%)

## Discussion

Decentralized screening and PCR testing using point-ofcare (POC) machines have been suggested as an innovative approach to help African countries scale up screening and testing for COVID -19 to improve the turnaround time for delivery of results.^9^ Using this mobile laboratory, the turn-around time for testing was on average three hours.

The actual time of testing of samples once it arrived at the mobile laboratory was approximately 2 hours, the extra one hour accounted for the time required for sample packaging, decontamination of carrier boxes once it arrives, and sample registration.

The rapidity and mobility of this laboratory in comparison with the average 3–4 days' turnover with the regular centralized PCR testing laboratories have many benefits (Table 3).

**Table 3:** Benefits of mobile laboratory testing for COVID-19 in Ghana

**Simple and easy to deploy on the field/ point of need**
**Allows for decentralization of COVID-19 testing even in resource** **limited settings in the field**
**Use of glove box obviates the need for a full bio-safety level 3** **(BSL3) laboratory**
**Long sample transportation time is eliminated**
**Reduces the workload on centralized laboratory**
**Rapid turnaround time for results**
**Facilitates early intervention for confirmed cases**
**Convenient for patients, and clinical/ public health decision** **makers**
**Greatly enhances contact tracing activities**
**May help reduce the anxiety associated with prolonged wait for** **results**
**Can be deployed to undertake confirmation of localized outbreaks** **of COVID-19**
**Can be deployed at the points of entry eg. airports and land borders** **to undertake rapid case confirmation**

Minimizing testing delays have the potential to minimize disease transmission by removing delays in contact tracing. With timely processing, persons requiring isolation can be readily isolated and contact tracing efforts implemented to forestall further disease spread within the community. The proportion of onward transmissions per index case that can be prevented depends on testing and tracing delays. In a recent modelling study, minimising testing delay had the largest impact on reducing onward transmissions 10. The proportion of onward transmissions per index case given a 0-day tracing delay ranged from up to 79·9% with a 0-day testing delay to 41·8% with a 3-day testing delay and 4·9% with a 7-day testing delay. This led the authors of that study to conclude that optimising testing and tracing coverage and minimising tracing delays, for instance with app-based technology, further enhanced contact tracing effectiveness, with the potential to prevent up to 80% of all transmissions. Similarly, it has been reported that shortening the waiting time for diagnosis can effectively decrease the basic reproduction number, significantly reduce the transmission risk, and effectively slow down the COVID-19 pandemic.^11^

Kucharski et al estimated that combined isolation and tracing strategies would reduce transmission more than mass testing or self-isolation alone.^10^

In that study, it was estimated that a high proportion of cases would need to self-isolate and a high proportion of their contacts to be successfully traced to ensure an effective reproduction number lower than one in the absence of other measures.

In combination with moderate physical distancing measures, self-isolation and contact tracing would be more likely to achieve control of SARS-CoV-2 transmission. Timely delivery of test results is therefore key to ensure early isolation of cases and tracing of contacts even as Ghana continues efforts in fighting against COVID-19.

The rapid turnaround time derived from the use of the mobile COVID-19 laboratory is convenient and was appreciated by patients, clinical/ public health decision makers and response teams, as it allowed rapid clinical decision-making regarding patient management. It has the potential to greatly enhance contact tracing activities and may help reduce the anxiety associated with prolonged wait for results. Patients whose test negative are spared unnecessary quarantine and the associated loss of work hours and income.

This rapid testing and release of results at the community level using the mobile testing approach can impact positively on the individual by reducing stigmatisation that may result from placing suspected cases in quarantine. Potentially, the country will also make savings on the financial costs emanating from prolonged care of persons who are placed in quarantine to await their test results; in this case, only persons who test positive are moved into isolation and cared for rather than quarantining large groups of community members.

The mobile van laboratory has a standby generator set while the mobile suitcase itself has a power pack that can store power for up to 24 hours. These features allow for the device to be deployed even in areas without electricity from the national grid including the most remote parts of the country. It can also function in times of power outages.

As a quality assurance measure, 20% of the 74 samples tested in the mobile laboratory were retested in the central laboratory at KCCR. The results showed 100% concordance same as the initial validation (Table 1) before deployment of the mobile laboratory. This indicates that the test procedure in the mobile laboratory was not inferior to that in the central reference laboratory. The 12% positivity rate reported in this study was a little lower but not significantly different from the national average of 14% from routine surveillance within the same time period.^12^,^13^

The WHO suggested a positivity rate of 3–12% as the general benchmark of adequate testing^14^ is consistent with our results. It is generally an accepted fact that one important way to understand if countries are testing sufficiently is to look at the share of tests returning the positive result (positive rate).

Ghana is not doing badly in comparison to other countries in the sub-region, our fluctuating positivity rate in and around 14% and delays in testing should be a cause for concern. The simple and easy to deploy mobile laboratory testing platform allows for the decentralisation of COVID-19 testing even in resource limited setting. This eliminates long sample transportation time and will reduce the workload on the centralized laboratory.

Potential use of the mobile COVID-19 laboratory described in this report is its deployment at Ghana's point-of-entry (eg. airports and land borders) to undertake rapid case confirmation and decision making. A partnership with the Customs Division of Ghana Revenue Authority (GRA) and Port Health Unit of Ghana Health Service might be useful to facilitate this effort. Recently, the Ghana Medical Association and other health professional groups have led a call for scaling up testing of frontline workers.^5^ The mobile laboratory can also be deployed within the hospital system to aid testing for health staff who meet the case definition for COVID-19 as this will help in quickly identifying staff with the infection and taking appropriate steps to isolate them thus breaking the chain of spread within the healthcare system.

The limitations to the use of the mobile laboratory system include working in a smaller space inside the laboratory, the size of the glovebox, and the portable PCR machine. As a result, samples have to be processed in smaller numbers and therefore important to prioritize the testing. Other challenges associated with maintaining the safety and security of personnel and equipment should not be taken lightly. Also, in identifying personnel for deployment to a hot-zone during a pandemic such as COVID-19, one should not only consider the training and expertise of an individual, but it is also important to consider the attitude and ability to adapt to the field conditions. In this project aside from the team from KCCR and GRA, biomedical scientists working in Obuasi were trained to assist with the testing.

## Conclusion

The mobile COVID-19 testing laboratory described in this report resulted in a rapid turnaround time for test results in the Obuasi municipality. Deployment of additional mobile laboratories can help to further minimize testing delays and positively impact contact tracing and disease transmission in Ghana.
